# Resumeq: A Novel Way of Monitoring Equine Diseases Through the Centralization of Necropsy Data

**DOI:** 10.3389/fvets.2019.00135

**Published:** 2019-04-26

**Authors:** Jackie Tapprest, Nathalie Foucher, Maud Linster, Eve Laloy, Nathalie Cordonnier, Jean-Philippe Amat, Pascal Hendrikx

**Affiliations:** ^1^Laboratory for Equine Diseases, French Agency for Food, Environmental and Occupational Health & Safety (ANSES), Goustranville, France; ^2^Pathological Anatomy Unit, National Veterinary School of Alfort, Maisons-Alfort, France; ^3^Coordination and Support Unit for Surveillance, ANSES, Lyon, France

**Keywords:** surveillance, mortality, equine, necropsy, resumeq

## Abstract

The French surveillance network for causes of equine mortality (Resumeq) was created in 2015 for the qualitative surveillance of equine mortality through the centralization in a national database of necropsy data and their subsequent epidemiological analysis. It was designed to identify the causes of equine mortality, monitor their evolution over time and space, and detect emerging diseases as early as possible. Resumeq is an event-based surveillance system involving various players and structures. It is organized around a steering body, a scientific and technical support committee and a coordination unit. Different tools have been developed specifically for Resumeq. These include standardized necropsy protocols, a thesaurus for the anatomopathological terms and the causes of equine death, and an interactive web application so that network contributors can display data analysis results. The four French veterinary schools, seventeen veterinary laboratories, and ten veterinary clinics already contribute to the production and centralization of standardized data. To date, the data from around 1,000 equine necropsies have been centralized. While most deaths were located in western France, the geographic coverage is gradually improving. Data analysis allows the main causes of death to be ranked and major threats identified on a local, regional or national level. Initial results demonstrate the feasibility and benefits of this national surveillance tool. Moreover, in the future, this surveillance could take an international dimension if several countries decided to jointly capitalize on their necropsy data.

## Introduction

It is necessary to monitor infectious animal diseases so as to describe their occurrence and help the planning, implementation and evaluation of risk mitigation actions (1, 2). The equine industry in France is vast, with an estimated one million equines and more than 30,000 breeders, 241 racecourses and 18,000 races each year (3). It provided about 180,000 jobs in 2017 (57,000 as the main activity) and generates around eleven billion euros per year. Equine imports and exports are numerous (respectively 10,000 and 18,000 in France in 2017). There are also frequent temporary movements of equines within Europe in the context of equestrian competitions or reproduction. In this situation, the quality of the health surveillance of the French horse industry is essential. In France, the surveillance networks monitoring equine diseases comprise various components, including compulsory notification of suspicions of regulated diseases (equine infectious anemia, West Nile fever, etc.) to the French Ministry of Agriculture's Food Directorate (DGAL), testing prior to sale directed by private partners, and surveillance of breeding stock managed by the French horse and riding institute (IFCE). There is also a voluntary passive surveillance system run by the RESPE (French network for epidemiological surveillance of equine diseases) veterinary association (4, 5). In order to complement these existing components, it appeared opportune to create a new surveillance network for major equine diseases (leading to death or euthanasia) through the centralization of necropsy data. Indeed, necropsy is recognized as an effective way of helping to monitor major diseases in animal populations (6–8). Moreover, in the equine sector, it is particularly important to monitor the causes of equine death through necropsy data because surveillance based on sanitary inspections at the slaughterhouse covers only a small part of the equine population, unlike other animal species mainly raised for consumption. Several studies relying on post-mortem examinations (9–14) have provided descriptive epidemiological data on the main equine diseases, thus helping to establish current disease trends in different populations and to suggest control and prevention measures. The ANSES Laboratory for Equine Diseases (ANSES-LPE) in Normandy, North West France, has for many years been a reference in the field of necropsies (11, 15–17). However, the surveillance data produced was limited to the Normandy region, and to obtain a national picture, it was necessary to change a regional system into a national system. The French surveillance network for causes of equine mortality (Resumeq) was created in 2015 for the qualitative surveillance of equine mortality by centralizing necropsy data in a national database that could be exploited for epidemiological purposes. The purpose of this event-based surveillance system is ultimately to provide the main health stakeholders with reliable epidemiological information on fatal enzootic or exotic equine diseases, and more particularly on contagious infectious diseases. The purpose of this research is to present the methodology used to construct the Resumeq network and the results obtained during its first three years of operation.

## Materials and Methods

### Objectives of the Network

The primary objective of Resumeq is to characterize the causes of mortality of necropsied equines and monitor their evolution over time and space. The second objective is to prioritize the causes of death. Another of its main objectives is the early detection of emerging exotic or new diseases.

### Organization

**Figure 1:**
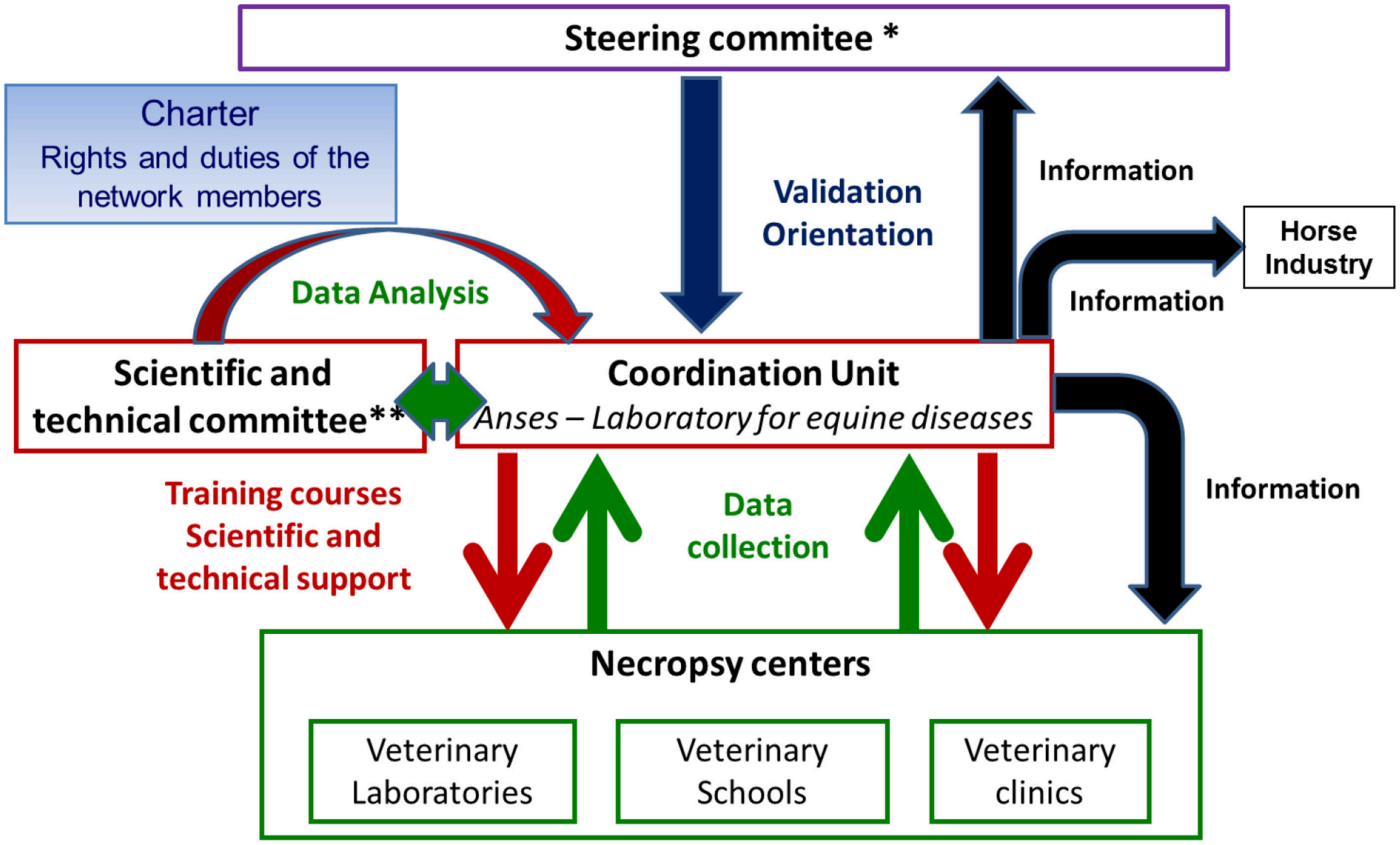
Organization of the French surveillance network for causes of equine mortality (Resumeq). ^*^Composition of the Steering Committee: ANSES (French Agency for Food, Environmental and Occupational Health & Safety), AVEF (French Equine Veterinary Association), DGAL (French Ministry of Agriculture's Food Directorate), ENVs (French veterinary schools), FNCC (National Federation of Horse Councils - organization which is the national representative of equine owners/holders in France), GDS France (National Federation of Sanitary Defense Groups), IFCE (French Horse and Riding Institute), CRN (Regional Council of Normandy), ADILVA (French Association of managers of public veterinary laboratories), GIS Centaure (scientific group for equine health), RESPE (French network for epidemiological surveillance of equine diseases), SNGTV (National Society of Veterinary Technical Groups). ^**^Composition of the Scientific and Technical Committee: L. Baudet, N. Cordonnier, A. Dadolle, N. Foucher, M. Foursin, P. Hendrikx, E. Laloy, M.N. Lemouland, M. Linster, C. Novella, M. Ogier de Baulny, E. Picard, E. Richard, J. Tapprest, I. Tourette, B. Vassiloglou.

Resumeq is an event-based surveillance system. Owners of equines can call on one of the members of the network (veterinary schools, veterinary laboratories or veterinary clinics) to carry out a post-mortem examination. The Resumeq network member performs the necropsy, recording and transmitting data to a coordination unit according to the protocols defined in the network framework. Due to the diversity of players and structures involved in this surveillance system, it has been organized on an institutional basis, with a steering body, a scientific and technical support structure and a coordination unit represented by ANSES-LPE (Figure 1). The scientific and technical committee includes different kinds of scientists: epidemiologists, pathologists and specialists of different equine diseases. The steering committee is made up of the various health stakeholders and of representatives of the equine industry. The stakeholders' involvement in the surveillance system is formalized in an operating charter.

### Data Standardization

Surveillance is based on the centralization of necropsy data in a national database managed by ANSES-LPE. The data must be of sufficient quality to be exploitable for epidemiological purposes. Surveillance is therefore based on harmonizing equine necropsy procedures nationwide, improving the skills of all network players, and standardizing the data collected.

#### Harmonizing Necropsy Procedures

Since the creation of the network in 2015, standardized necropsy procedures have been defined by dedicated working groups taking into account the variability of conditions in which the different players perform necropsies, as well as the age categories. Complete protocols taking into account the cadaver position (dorsal recumbency, lateral recumbency or dead animal hanging on a hoist) have been developed for the veterinary schools and laboratories.

These complete protocols systematically include external examination of the cadaver, examination of the subcutaneous connective tissue, muscles and joints, evisceration, and examination of the abdominal organs, evisceration and examination of the thoracic organs, isolation and examination of the upper respiratory tract, cervical esophagus, thyroid gland and tongue. Depending on the epidemiological and clinical context, various complementary options are also possible: collection of cerebrospinal fluid, isolation of the head and examination of the brain, guttural pouches and nasal cavities, examination of the spine, and a sagittal section of the hooves. Due to the difficulty of performing a complete post-mortem examination in the field, veterinary practitioners may furthermore use a partial protocol focusing on a specific anatomical region. In any case, the nature of the necropsy protocol used must be specified by the Resumeq members when entering data in the database. Finally, a specific procedure for necropsy examination in the context of abortions and stillbirths has been developed which is valid for all players.

#### Training Resumeq Members

At the same time, emphasis was placed on the initial and ongoing training of network members through the regular organization of training courses and internships to improve their expertise in the diagnosis of the causes of equine mortality. These target not only current network participants, but also potential future members through a partnership with veterinary schools, which propose students who have specialized in the equine sector and/or in pathological anatomy. Short training sessions of between one and three days are organized annually for network members by the coordination unit (ANSES-LPE) and the pathological anatomy unit of the National Veterinary School of Alfort (ENVA). They include practical and theoretical training designed to improve the skills of players in terms of implementing equine necropsy procedures and diagnosing the causes of mortality, as well as entering data in the national database. The different training sessions are geared to the different skill levels of the Resumeq players. Members of the network who cannot demonstrate a specific competence in post-mortem examination of equines must undergo a mandatory initial training course soon after joining the network. Finally, the coordination unit offers constant scientific and technical support. Indeed, network members regularly call on the coordination unit during or after a necropsy for advice on necropsy procedures, sampling techniques, the interpretation of observed abnormalities or guidance on additional examinations to be performed to clarify the etiology and especially to protect the herd from which the necropsied equine came.

#### Standardizing Collected Data

The essential data to be collected to meet network objectives were selected by a dedicated working group and then subjected to a standardization process. Data considered to be essential are those on epidemiological context, animal identification, those related to space and time, summarized clinical data, necropsy data needed to qualify a cause of death, a confidence index for the cause of death, and data on supplementary examinations. The most advanced standardization work involved data on the lesion table and causes of death. Thus, two thesauruses dedicated to equines have been developed: a thesaurus of anatomopathological terms and a thesaurus of equine mortality causes. The anatomopathological thesaurus is divided into two parts. The first part, concerning the anatomical location, includes three levels and 111 items. The first level consists of the different anatomical apparatus (cardiovascular system, digestive system, etc.); the second level corresponds to a first degree of detail on the anatomical location (heart, vessels, small intestine, etc.); and the third level to an even finer degree of location (epicardium, myocardium, duodenum, jejunum, etc.). The second part, concerning lesions, includes three levels and 125 items. The first level corresponds to the category of lesion (morphological anomaly, inflammatory lesion, mechanical and traumatic lesion, etc.), the second level corresponds to a first degree of precision within the lesional categories (ulcerative inflammatory lesion, fibrinous inflammatory lesion, etc.) and the third level to an additional degree of precision (perforative ulcerative inflammatory lesion, stenosing ulcerative inflammatory lesion, etc.). The thesaurus of anatomopathological terms was derived from the reference thesaurus set up by the French SAGIR surveillance network of wildlife mortality (18), with members' approval. The thesaurus was then tailored to equines with the help of ENVA. The cooperation between two mortality surveillance networks focusing on different specie offers contributors to both networks a single anatomopathological reference system, thus improving efficiency and quality when entering their data. A glossary for the anatomopathological thesaurus was then developed by ENVA's pathological anatomy unit to help players entering lesional data. Use was furthermore made of ANSES-LPE's experience in diagnosing causes of equine mortality and its own database, which contains necropsy data going back more than 20 years, to classify the causes of mortality according to different main and secondary categories, and led to the creation of a thesaurus dedicated to the causes of equine mortality. This thesaurus is organized differently according to the age category. For fetuses and foals <1 month old, the first level corresponds to a classification according to two categories: “infectious” or “non-infectious.” In the “infectious” category, the second level corresponds to the types of infectious causes (placentitis, septicemia, meningitis, etc.) and the third and fourth levels are used to detail the type of infectious agent (bacterial, viral, mycotic) and its precise designation. In the “non-infectious” category, the second level details directly the different causes of abortion or mortality of the young foal (twin pregnancy, umbilical cord twist, vesical rupture, etc.). For equines older than 1 month, the first level is used for classification according to the anatomical system mainly affected (respiratory system, musculoskeletal system, systemic, etc.). Within each anatomical system, the second level corresponds to a classification according to two categories: “infectious” or “non-infectious.” In the “infectious” category, the third level corresponds to the types of infectious causes (interstitial pneumonia, typhlocolitis, myeloencephalopathy, etc.), then the fourth and fifth levels are used to detail the type of infectious agent (bacterial, viral, mycotic) and its precise designation. In the “non-infectious” category, the third level corresponds to a classification according to the types of causes of mortality (tumoral, mechanical, toxic, etc.). The fourth and fifth levels finally offer more details (lymphosarcoma, cervical compressive myelopathy, volvulus nodosus, etc.). In total, the thesaurus includes 504 items (Supplementary Tables 1, 2). This thesaurus is designed to evolve, it being possible to add new causes of mortality according to future needs.

### Centralizing and Consolidating Data

A Web interface is used by Resumeq contributors to enter their data directly in the dedicated national database. The vast majority of data is entered through drop-down menus. The lesional data and cause of death in particular are entered using drop-down menus applicable to both thesauri in order to obtain standardized data. These data are then consolidated centrally. Partially automated data quality control has been set up to retrieve a list of irregularities on a monthly basis. The quality control is carried out through a dedicated script using R software (19). This provides information about missing data for all the interface fields and makes it possible to carry out checks for likelihood and coherence (length of the transponder number, date of death < date of necropsy, etc.). The coordination unit (ANSES-LPE) often has to investigate these glitches and discuss them individually with the contributors concerned so that they may be corrected whenever possible. This time-consuming step of data consolidation is essential because not only is quality of data input improved, but these regular discussions with network members contribute to their training and boost their motivation.

### Communication and Adding Value to Data

Data are regularly analyzed by the coordination unit (ANSES-LPE) and the scientific and technical support structure to identify the main causes of mortality in equines according to different age categories. Tools to communicate results and coordinate network activities were also set up, with the creation of a website (https://sites.anses.fr/fr/minisite/resumeq) and a Web interface developed using the R shiny application (19) that allows the interactive display of network results. By regularly updating this interface, players have the very latest feedback, which fosters network dynamics. A biannual newsletter is also published to communicate main network facts and figures, interviews with members and activities, for example. When a potential hazard is identified at a regional or national level, Resumeq uses different channels to disseminate alerts, including the epidemiological surveillance network for equine pathology (RESPE) (20) for dissemination to veterinarians and the French Horse and Riding Institute (IFCE) (21) for widespread dissemination throughout the French equine sector.

### Operating Indicator Dashboard

To help steer Resumeq, a complete, specific set of network operating indicators was developed based on work achieved by the French ESA (epidemiological surveillance in animal health) Platform (22, 23). They take into account the various activities and characteristics of the surveillance system: management and coordination, data collection and management, feedback and communication, training, network coverage, and member contributions. They all have a quantified objective that can evolve over time according, in particular, to Resumeq's development. These indicators were discussed by the Scientific and Technical Committee and validated by the Steering Committee at the end of 2017. In particular, to define the targeted value of the proportion of dead equines necropsied by Resumeq, the Scientific and Technical Committee started from the maximum percentage of dead equines necropsied per year in a *département* in France (10%). This maximal value was found for the *département* of Calvados where ANSES-LPE is located. This value was considered as an ideal long-term goal, the medium-term goal being 5%. Given that Resumeq is a young network that does not have members in each of the French *départements*, it seemed reasonable for the members of the committee to set the 2017 target at 1.5%. The 19 main indicators are presented in Table 1.

**Table 1:** The 19 main network operating indicators for Resumeq: definitions, quantified objectives and target achivement rates for 2017.

**Category and definition of the performance indicators**	**Expected value for 2017**	**Results for 2017**	**Target achievement rates for 2017**
**STEERING COORDINATION**			
Number of national meetings	2/year	2	100%
Number of local meetings	4/year	6	>100%
**FEEDBACK COMMUNICATION**			
Number of newsletters published	2/year	1	50%
Number of results reviews held	1/year	1	100%
Compliance with the monthly updating of results in R shiny	>90%	Not applicable in 2017	Not applicable in 2017
Number of written/oral communications on network operation	2/year	5	>100%
**NETWORK COVERAGE AND REPRESENTATIVENESS**			
Number of new members	1 VL and 2 VCs/year	5 VLs and 5 VCs	>100%
Proportion of necropsy centers [VS-VL]: number of Resumeq necropsy centers / number of potential necropsy centers in France	2017: ≥50%	22/39 (56%)	100%
Proportion of active Resumeq necropsy centers [VS-VL]:number having entered necropsy data / total number	≥80%	11/22 (50%)	63%
Proportion of active Resumeq veterinary clinics: number having entered necropsy data / total number	≥ 20%	2/10 (20%)	100%
Proportion of *départements* “under surveillance”: number of *départements* with necropsy cases / total number	≥80%	58/96 (60.4%)	75.5%
Proportion of dead equines necropsied by Resumeq: number of necropsy cases / number of equines removed by a fallen stock company in 2017	1.5%	287/37,816 (0.76%)	50%
**COLLECTION AND DATA MANAGEMENT**			
Proportion of necropsies whose data entry began less than 15 days after the necropsy	≥60%	57%	95%
Proportion of necropsies with a cause of death completely filled in	≥95%	98.5%	100%
Proportion of equines with a known *département* of death	≥95%	99%	100%
Proportion of equines with a known *département* during life	≥90%	97%	100%
Proportion of equines with an identification number entered in the database	≥90%	90%	100%
**TRAINING**			
Number of training sessions organized	1	1	100%
Proportion of VSs and VLs that have followed at least one training session within two years of joining Resumeq	≥80%	80%	100%

*(VS, veterinary school; VL, veterinary laboratory; VC, veterinary clinic)*.

## Results

### Surveillance Review

**Figure 2:**
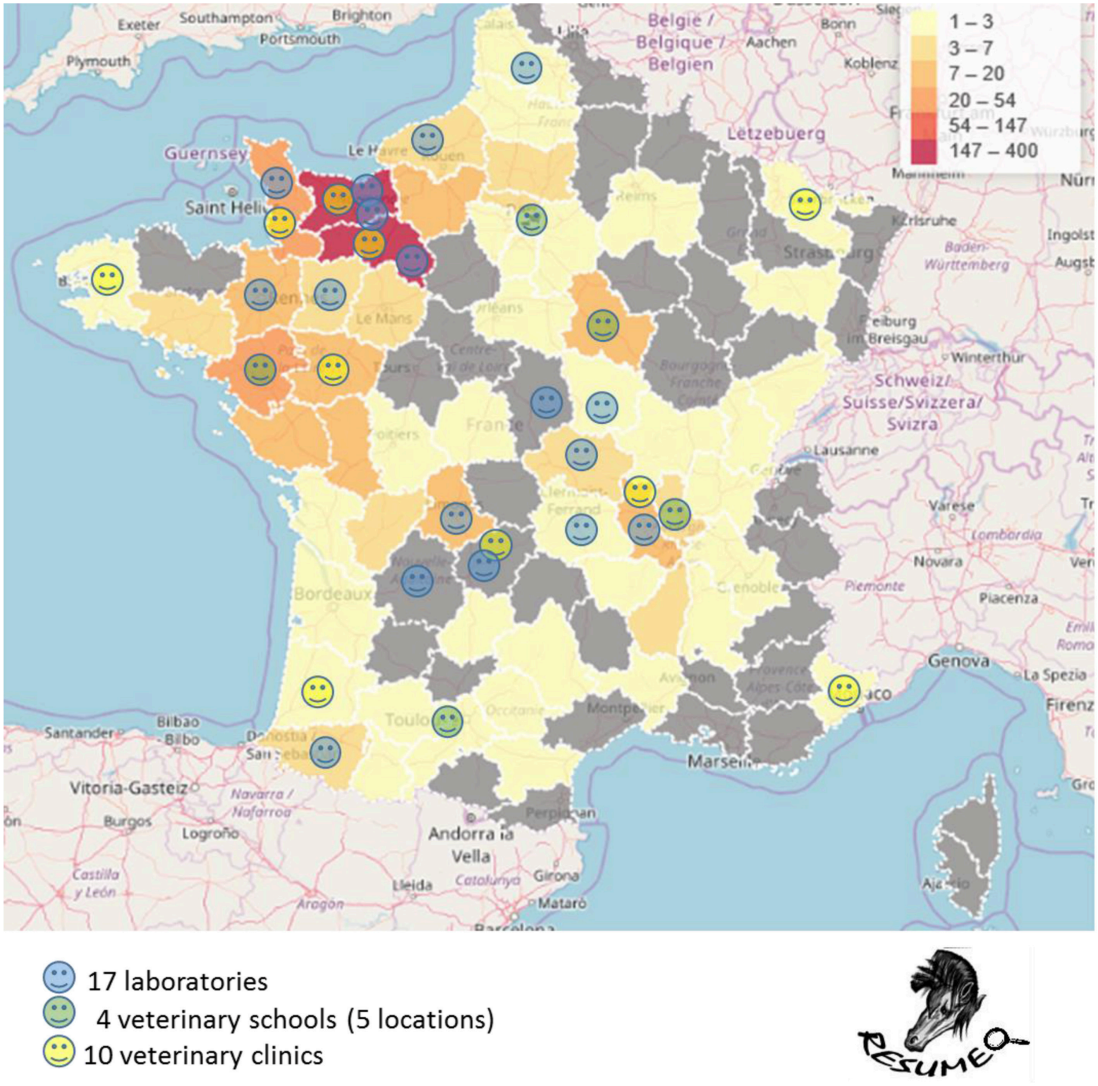
Geographic distribution of the 32 Resumeq network members (colored smileys) and of the number of necropsied equines by *département* from 2015 to 2017 (color scale).

**Figure 3:**
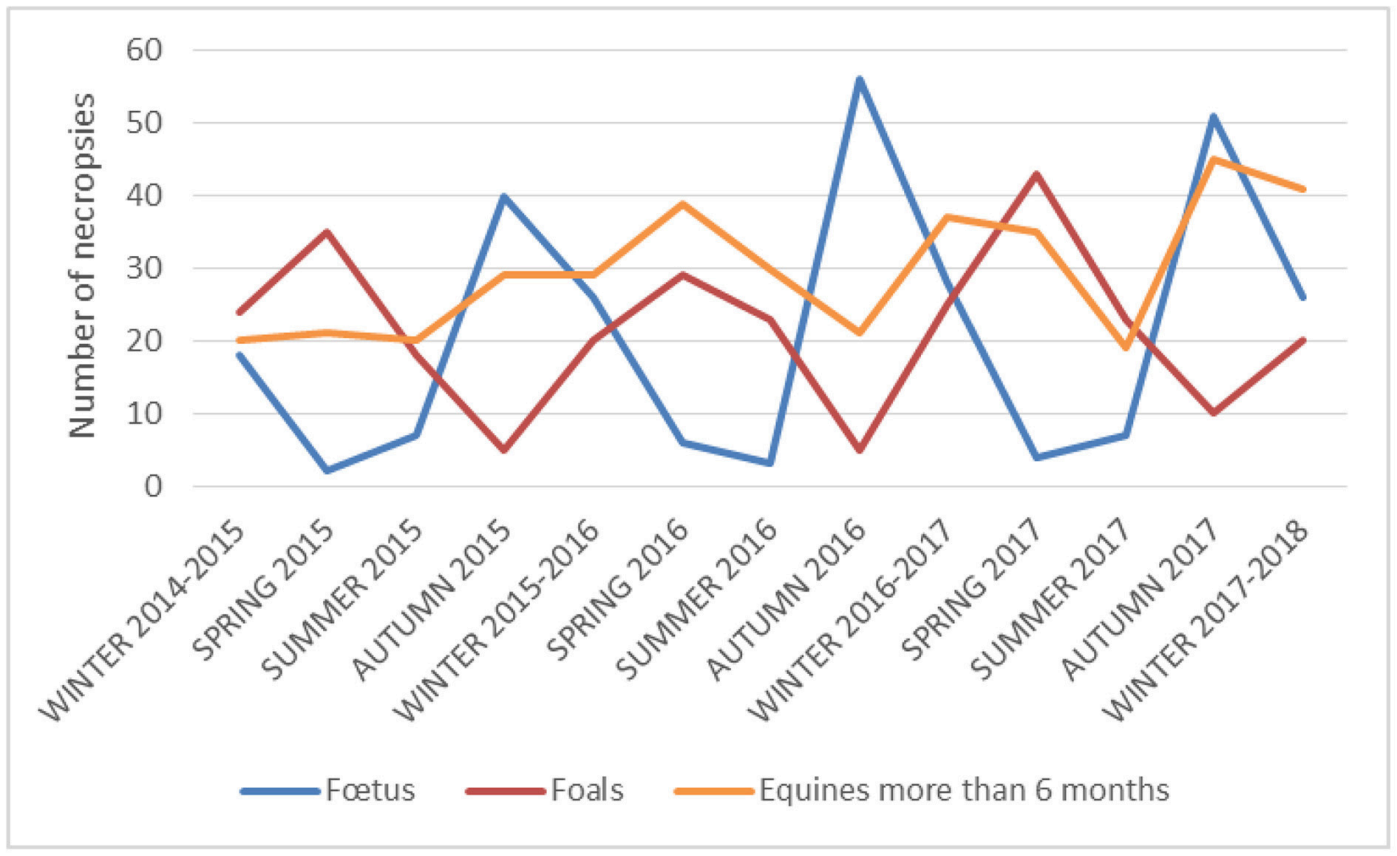
Temporal variation of equine necropsies (2015–2017) according to age categories.

**Figure 4:**
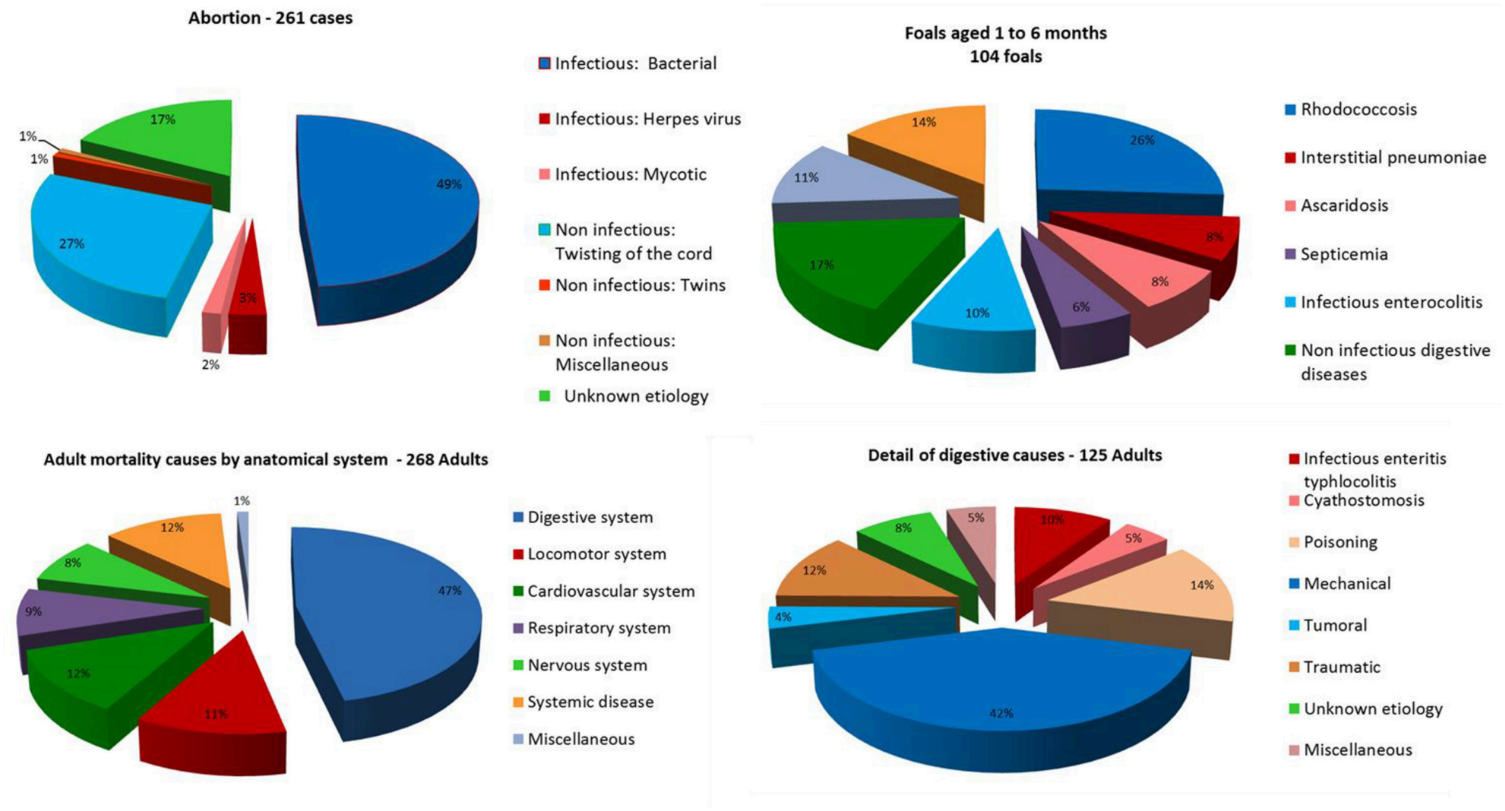
Distribution of the causes of death of equines in different age categories (2015–2017).

The four national veterinary schools (in five locations), seventeen veterinary laboratories and ten veterinary clinics already contribute to the production and centralization of standardized data (Figure 2). To date, data from approximately 1,000 necropsies performed since 2015 on dead equines from 58 *départements* (French administrative units) out of 96 French metropolitan *départements* have been collected. Most come from *départements* in the West of France, but geographic coverage is gradually improving (Figure 2). The temporal variation of necropsy submissions according to different age categories is presented in Figure 3. For fetuses, the number of necropsies peaked in autumn and a minimum was reached in spring. Most foal necropsies are performed during spring. Finally, for equines older than 6 months, the number of necropsies is more constant throughout the year. Data analysis makes it possible to prioritize the main causes of mortality in equines according to different age categories (Figure 4). As far as abortions are concerned, there is a predominance of bacterial placentitis (49%) among the infectious causes of abortion. Although many different germs are involved, there is a higher frequency of beta hemolytic streptococci (45%), staphylococci (16%) and different enterobacteria (*Klebsiella pneumoniae* (8%), *Escherichia coli* (15%), etc.). Abortions due to herpes virus (types 1 and 4) are much less frequent (3%), but they need to be identified because of their contagious nature and therefore the threat they represent for all other equines on the stud farm. An analysis of the first results for foals aged from 1 to 6 months confirms the prominent place of rhodococcosis (26%) among the causes of mortality, with a variety of lesional forms (pulmonary, digestive, osteoarticular, mixed) even though suppurative bronchopneumonia is the most common form (17, 24, 25). However, there appears to be an increasing number of foals with interstitial pneumonia causing acute respiratory distress syndrome (8%) in recent years. In adult equines, results show the prominence of digestive diseases (47%) among the causes of mortality, with Resumeq also monitoring cases of digestive salmonellosis. Even though neurological disorders represent only 8% of the causes of adult mortality, they are of particular importance because of the infectious agents of very high concern that they involve, including herpes virus type 1 or West Nile virus. In this regard, during the West Nile fever epizootic in south-eastern France in 2015, it was the mobilization of the Resumeq network that enabled the virus to be isolated, sequenced, and phylogenetically analyzed (26) following the necropsy of a dead equine. Apart from West Nile fever, Resumeq has made it possible to objectify other threats at a local or regional level. This has regularly been the case, for example, through the identification of contagious causes of abortion such as Rhinopneumonitis or the identification of toxic environmental causes such as in Fall 2015, when several cases of fatal intoxication by acorns were identified in the western part of France. The dissemination of alerts through different channels in order to transmit the information to veterinary practitioners and the horse industry represents a potential way of limiting the number of deaths associated with identified threats such as these.

### Operating Review

Resumeq's main operating indicators and the percentages of achievement of the objectives set for 2017 are presented in Table 1. Indicators were considered satisfactory when their values for 2017 reached or exceeded the expected value defined by the Scientific and Technical Committee and validated by the Steering Committee. The indicators used to monitor the network's management, coordination, and training activities are all satisfactory. Feedback and communication were subject to focused developments in 2017, in particular with the creation of a Web interface rolled out in early 2018. The indicator relating to the updating of results via this interface could not be evaluated for 2017 but the monthly update rate target for 2018 has so far been met. The delay in the publication of the second network newsletter explains the low value of the corresponding indicator but has had no effect on network operation. The data quality indicators (proportion of equines with an identification number entered in the database, proportion of equines with a known *département* during life, proportion of equines with a known *département* of death, proportion of necropsies with a fully filled in cause of death) are very satisfactory, whereas the value of the indicator relating to the delay prior to data being entered in the database (proportion of necropsies whose data were entered <15 days after the necropsy) is unsatisfactory. The value of the indicators relating to network coverage and representativeness in terms of causes of death seem insufficient. In fact, the number of new members is very satisfactory and far exceeds the target set for 2017. The proportion of necropsy centers (veterinary schools or veterinary laboratories) subscribing to Resumeq is reasonable, but the values decrease when focusing on the number of active necropsy centers, that is to say centers having entered necropsy data during 2017. At the same time, the proportion of *départements* under surveillance (58/96) is slightly lower than the expected values in 2017. Finally, the national proportion of dead equines necropsied by Resumeq (compared to the total number of equines removed by a fallen stock company) is lower than the expected values in 2017, and the proportions of dead equines necropsied by Resumeq by *département* varied from 0% to 11%.

## Discussion

Resumeq's initial results are very encouraging. Data analysis allows the main causes of mortality in equines to be ranked and major threats identified on a local, regional or national level. Currently, however, the sample of equines necropsied through Resumeq is not representative of the population of dead equines in France. It has nevertheless made it possible to objectify certain abnormal events despite the network's short operating time (3 years so far). It is essential to continue improving Resumeq's geographic coverage for two main reasons. The first objective is to be representative of the national population of dead equines in order to reflect current trends in the causes of equine mortality in France. The second objective is to be present in particular areas with a higher risk, such as the South-East of France for West Nile fever, so as to be truly capable of detecting the potential emergence or re-emergence of certain diseases. Since Resumeq is a non-specific surveillance network, many different diseases, syndromes or themes can benefit from its results and can be monitored over time and space. For example, in both foals and adults, the main parasitic causes of mortality (ascariasis and larval cyathostomosis, respectively) can be tracked over time and space, and Resumeq results used as indicators to monitor the possible development of resistance to anthelmintics. Data analysis can also help identify research topics. For example, the increasing number of dead foals with interstitial pneumonia in recent years shows that this lesional entity, whose etiology remains uncertain (27), deserves further investigation through a non-specific search for pathogens. Finally, apart from known causes of mortality, it could be interesting to look at the proportion represented by cases of mortality with an unknown etiology. The failure to identify the cause may be related to various factors, including the absence of fetal appendices in the case of an abortion, poor conservation of the cadaver, or the impossibility of carrying out additional examinations needed to clearly demonstrate the etiological agent. However, it could be important to initiate monitoring of the proportion of unclear causes of mortality since any unusual increase could potentially correspond to the emergence of a new etiology (7). However, in the context of setting up such syndromic surveillance, it would probably be preferable to separately evaluate data from veterinary schools and veterinary laboratories (which carry out a complete necropsy protocol and have greater expertise) from data from practicing veterinarians who implement partial protocols and whose level of expertise in the field of necropsy is very variable.

Despite the few years that Resumeq has been running, the temporal description of necropsy submissions has revealed a marked seasonality for fetuses and foals. The period with the highest number of dead foals submitted for necropsy is logically superimposed on the foaling season (increase in the population at risk). Similarly, the lack of fetuses submitted for necropsy in spring can be explained by a reduced number of pregnant mares during this period. These results are consistent with a previous study showing a similar seasonality in equine deaths in France through the temporal description of data from rendering plants, which are centralized in the Fallen Stock Data Interchange (FSDI) database, managed by the French Ministry of Agriculture (28). In the future, it would be useful to concomitantly monitor the temporal evolution of the total number of dead equines in France through the FSDI database and that of the number of necropsies submitted to the Resumeq network. This combined follow-up could be used to explore how the two sources of mortality data could complement and potentiate each other in order to improve knowledge and surveillance of equine mortality in France. The network is recent and only operating indicators have been implemented so far. It is nevertheless necessary and planned to set up health indicators as well as thresholds defining an alert. Reflection on these indicators will require sufficient history in terms of data / results and will be conducted within the Scientific and Technical Committee. For well-defined diseases, determination of health indicators will be based on work achieved by the French ESA Platform (29). Better knowledge of the epidemiological situation (number of cases/outbreaks, spatial and temporal evolution) will then make it possible to implement appropriate prevention and control measures. For syndromic surveillance, the network being young and the number of necropsies increasing from 1 year to the next, it will be necessary to wait until the number of necropsies stabilizes before being able to study quantitatively time series. As there is an extensive list of statistical methods available for the early detection of an abnormal signal, it would then be necessary to compare and choose the best algorithm for event detection (30).

Most indicators used to monitor the network are satisfactory. The data quality indicators are particularly satisfactory and these good results can be explained by the emphasis placed on the data entry training offered to Resumeq members and by the checking and consolidation of the data collected through individual discussions with network contributors. On the other hand, the delay prior to data being entered in the database is too long. To reduce this delay, we need to raise the awareness of Resumeq members about the importance of the database as a channel for rapid feedback to the coordination unit. Currently, Resumeq members quickly contact the coordination unit (ANSES-LPE) directly if they come across an abnormal event, and only complete the database afterwards. The delay in data entry does not therefore affect the possibilities of an alert for well-identified individual events. On the other hand, reducing the delay between completion of the necropsy and entry of the corresponding data will be a major point to improve for syndromic surveillance development in the short and medium term (follow-up of unknown etiologies, or of clinical and/or pathological syndromes). It is the average value of the indicators relating to network coverage and representativeness that guide the priority efforts to be undertaken. Indeed, since 2015, Resumeq has experienced a particularly rapid development with 32 members to date. This development has continued throughout 2017, with a very satisfactory number of new memberships and a concomitant improvement in geographic coverage. However, with the exception of a few members, the number of equine necropsies performed per contributor remains low. Indeed, the four national veterinary schools and only a few laboratories perform most of the necropsies collected by Resumeq. The other Resumeq members perform <5 to ten equine necropsies per year. Several factors are involved, notably the difficulties in transporting dead equines to necropsy centers, the impossibility in the vast majority of cases for practicing veterinarians to carry out an equine necropsy at the rendering enclosure, the cost of the necropsy, and the reluctance of some equine owners to have a necropsy performed on their animal. The lack of training among practicing veterinarians and laboratories in necropsy techniques and the diagnosis of causes of equine mortality is also a limiting factor. By monitoring these operating indicators, we can thus identify priority areas of work, such as the initial and ongoing training of players, the search for solutions to the cadaver transport issue, and regular feedback to members in order to boost and keep up their motivation (31).

Resumeq's initial results demonstrate the feasibility and value of the surveillance of the causes of equine mortality nationally. The network must now be consolidated, notably by increasing the number of necropsies performed by each of its members, and extending its coverage to the whole of France. In the future, however, this surveillance could take on an international dimension if several countries decided to fully exploit their necropsy data in partnership. The potential development of an international network has to be considered and built up over the long term. Indeed, many advantages can be envisaged, including an overview of the variations in the main equine diseases between countries and the alert capability such a network could provide at an international level. On the other hand, many difficulties /constraints have to be resolved first, and in particular major data issues (data property, data access, data confidentiality, etc.) and the need to adopt a common/similar standardization regarding data and necropsy protocols. The development of data standard and interoperability is currently a huge topic in the veterinary field. In particular, several ontologies are being elaborated in order to facilitate the development of smart system for data use in health surveillance (32) and the creation of thesauri can also contribute to this approach. In the present case, the two thesauri dedicated to the surveillance of equine mortality developed within Resumeq can partially solved the issue represented by the lack of common data standard among countries. Indeed, given the fact that no thesaurus of anatomopathological terms and no thesaurus of mortality causes were previously published on equines, those thesauri represent a first and important step toward standardization and should facilitate the extension of the Resumeq network. In particular, adaptation/translation of these thesauri could represent a first topic of collaboration between countries.

## Author Contributions

JT, NF, J-PA, NC, and PH contributed to the conception/construction of the network and of the monitoring tools. NF, ML, EL, and NC contributed to data collection. JT, NF, J-PA, ML, EL, NC, and PH contributed to data analysis. JT wrote the manuscript with support from NF and PH and all authors validated the work before submission.

### Conflict of Interest Statement

The authors declare that the research was conducted in the absence of any commercial or financial relationships that could be construed as a potential conflict of interest.
