# Supplementary material for: Resumeq: A Novel Way of Monitoring Equine Diseases Through the Centralization of Necropsy Data
Source: Front Vet Sci. 2019 Apr 26;6:135. doi: 10.3389/fvets.2019.00135 (PMC6524722; doi:10.3389/fvets.2019.00135)
Supplement: Supplementary file 1 [file Data_Sheet_1.PDF]

**Supplementary Table 1**

| RESUMEQ NETWORK | | | |
|---|---|---|---|
| **Thesaurus of the causes of equine mortality** | | | |
| Age categories: Fetus, stillborn, ]0 - 24h], ]24h - 1 month] | | | |
| fetus | infectious | macroscopic placentitis | bacterial |
| | | | mycotic |
| | | | bacterial and mycotic |
| | | | unidentified pathogen |
| | | | no search for pathogen |
| | | absence of macroscopic placentitis | bacterial |
| | | | unidentified pathogen |
| | | | no search for pathogen |
| | | | viral (not herpes) |
| | | Rhinopneumonitis | EHV1 |
| | | | EHV4 |
| | | Leptospirosis | |
| | | absence of placenta | bacterial |
| | | | unidentified pathogen |
| | | | no search for pathogen |
| | | | viral (not herpes) |

| | | | |
|---|---|---|---|
| | non infectious | umbilical cord twist | |
| | | hydrops of fetal membranes | |
| | | placental insufficiency | |
| | | premature detachment of the placenta | |
| | | twin pregnancy | |
| | | pregnancy in the placental body | |
| | | strangulation by the umbilical cord | |
| | | congenital abnormalities | |
| | | traumatic | |
| | | other | |
| | | abnormal umbilical cord | |
| | unknown etiology | | |

| | | | |
|---|---|---|---|
| stillborn | infectious | macroscopic placentitis | bacterial |
| ]0 - 24h] | | | mycotic |
| | | | bacterial and mycotic |
| | | | unidentified pathogen |
| | | | no search for pathogen |
| | | absence of macroscopic placentitis | bacterial |
| | | | viral (not herpes) |
| | | | unidentified pathogen |
| | | | no search for pathogen |
| | | Rhinopneumonitis | EHV1 |
| | | | EHV4 |
| | | Leptospirosis | |
| | | absence of placenta | bacterial |
| | | | unidentified pathogen |
| | | | no search for pathogen |
| | | | viral (not herpes) |
| | | | |

| | | | |
|---|---|---|---|
| | non infectious | umbilical cord twist | |
| | | hydrops of fetal membranes | |
| | | placental insufficiency | |
| | | premature detachment of the placenta | |
| | | twin pregnancy | |
| | | pregnancy in the placental body | |
| | | strangulation by the umbilical cord | |
| | | congenital abnormality | |
| | | anoxia/asphyxia | |
| | | premature birth/ dismaturity | |
| | | premature rupture of the umbilical cord (hemorrhage) | |
| | | fracture of the skull | |
| | | fracture of the ribs (hemorrhage) | |
| | | other | |
| | | tamponade | |
| | | dystocia | |
| | unknown etiology | | |

| | | | |
|---|---|---|---|
| ]24h- 1 week] | infectious | septicemia | bacterial |
| ]1 week- 1 month] | | | viral (not herpes) |
| | | | unidentified pathogen |
| | | | no search for pathogen |
| | | Rhinopneumonitis | EHV1 |
| | | | EHV4 |
| | | bronchopneumonia-pneumonia | no search for pathogen |
| | | | unidentified pathogen |
| | | | bacterial |
| | | | |
| | | | viral (not herpes) |
| | | | |

| | | | |
|---|---|---|---|
| | | enteritis | bacterial |
| | | | viral |
| | | | no search for pathogen |
| | | | unidentified pathogen |
| | | enterocolitis | bacterial |
| | | | viral |
| | | | unidentified pathogen |
| | | | no search for pathogen |
| | | typhlocolitis | bacterial |
| | | | viral |
| | | | unidentified pathogen |
| | | | no search for pathogen |
| | | colitis | bacterial |
| | | | viral |
| | | | unidentified pathogen |
| | | | no search for pathogen |
| | | meningitis | bacterial |
| | | | viral |
| | | | unidentified pathogen |
| | | | no search for pathogen |
| | | osteomyelitis | bacterial |
| | | | unidentified pathogen |
| | | | no search for pathogen |
| | | polyarthritis | bacterial |
| | | | unidentified pathogen |
| | | | no search for pathogen |
| | | myositis | bacterial |
| | | | unidentified pathogen |
| | | | no search for pathogen |
| | | Tyzzer's disease | |

| | | | |
|---|---|---|---|
| | non infectious | anoxia/asphyxia | |
| | | premature / immaturity | |
| | | isoerythrolysis in neonatal foal | |
| | | congenital abnormality | |
| | | perforating ulcer | |
| | | tamponade | |
| | | hemorrhage | |
| | | gastrocnemius muscle rupture | |
| | | anaphylatic shock | |
| | | fracture of the skull | |
| | | fracture of the ribs (hemorrhage) | |
| | | other | |
| | | acute pulmonary edema | |
| | | nutritional myopathy | |
| | | rupture of the urinary bladder | |
| | | polytraumatism | |
| | | intussusception of the small intestine | |
| | unknown etiology | | |
