# Supplementary material for: Resumeq: A Novel Way of Monitoring Equine Diseases Through the Centralization of Necropsy Data
Source: Front Vet Sci. 2019 Apr 26;6:135. doi: 10.3389/fvets.2019.00135 (PMC6524722; doi:10.3389/fvets.2019.00135)
Supplement: Supplementary file 2 [file Data_Sheet_2.PDF]

Supplementary Table 2

# RESUMEQ NETWORK

## Thesaurus of the causes of equine mortality

### Age category : more than 1 month old

| | | | | |
|---|---|---|---|---|
| respiratory system | infectious | sinusitis | bacterial | |
| | | | mycotic | |
| | | | viral | |
| | | | unidentified pathogen | |
| | | | no search for pathogen | |
| | | pulmonary rhodococcosis | bacterial | |
| | | | unidentified pathogen | |
| | | | no search for pathogen | |
| | | other bronchopneumonia pneumonia | bacterial | |
| | | | mycotic | |
| | | | bacterial and mycotic | |
| | | | viral | |
| | | | no search for pathogen | |
| | | | unidentified pathogen | |
| | | pleuropneumonia | bacterial | |
| | | | mycotic | |
| | | | bacterial and mycotic | |
| | | | viral | |
| | | | unidentified pathogen | |
| | | | no search for pathogen | |
| | | guttural pouch mycosis (hemorrhage) | yeast and fungal | |
| | | | unidentified pathogen | |
| | | | no search for pathogen | |
| | | guttural pouch empyema | bacterial | |
| | | | unidentified pathogen | |
| | | | no search for pathogen | |
| | | compression of trachea by abcesses | bacterial | |
| | | | unidentified pathogen | |
| | | | no search for pathogen | |
| | | other | | |
| | | interstitial pneumonia | | |

| | | | | |
|---|---|---|---|---|
| | non infectious | tumoral | tumor of sinuses | |
| | | | pulmonary tumor | |
| | | | other | |
| | | allergic | laryngeal oedema | |
| | | | chronic obstrutive pulmonary disease | |
| | | | other | |
| | | traumatic | hanging | |
| | | | pneumothorax following rib fracture(s) | |
| | | | rupture of the diaphragm | |
| | | | bronchopneumonia following intubation | |
| | | | other | |
| | | | compression of the upper respiratory tract | |
| | | | penetrating wound to the thoracic wall | bullet |
| | | | | object |
| | | | | horn |
| | | congenital or hereditary | diaphragmatic hernia | |
| | | | other | |
| | | mechanichal | aspiration pneumonia | |
| | | | obstruction of the upper respiratory tract by a foreign body | |
| | | | drowning | |
| | | | other | |
| | | | tracheal collapse | |
| | | toxic | inhalation of fire fumes | |
| | | | other | |
| | | unidentified | | |
| | | other | transport pneumonia | |
| | | | acute pulmonary edema | |
| | | | pulmonary fibrosis | |
| | parasitic | Dictyocaulus | | |
| | | other | | |
| | unidentified | | | |
| | other | | | |

| | | | | |
|---|---|---|---|---|
| gastrointestinal system | infectious | endotoxemia or enterotoxemia | bacterial | |
| | | | unidentified pathogen | |
| | | | no search for pathogen | |
| | | enteritis | bacterial | |
| | | | unidentified pathogen | |
| | | | no search for pathogen | |
| | | typhlocolitis | bacterial | |
| | | | unidentified pathogen | |
| | | | no search for pathogen | |
| | | digestive rhodococcosis | bacterial | |
| | | | unidentified pathogen | |
| | | | no search for pathogen | |
| | | other | | |
| | non infectious | tumoral | lymphosarcoma (or lymphoma) | |
| | | | other | |
| | | | adenocarcinoma | |
| | | congenital or hereditary | megaesophagus | |
| | | | diaphragmatic hernia | |
| | | | inguinal hernia | |
| | | | Meckel's diverticulum | |
| | | | other | |
| | | | cleft palate | |
| | | | umbilical hernia | |
| | | traumatic | diaphragm rupture | |
| | | | oesophagus-perforation | |
| | | | caecum - tear | |
| | | | ascending colon - tear | |
| | | | rectal laceration | |
| | | | other | |
| | | | small intestine - tear | |
| | | | penetrating wound to the abdominal wall | bullet |
| | | | | object |
| | | | | horn |
| | | mechanichal | primary gastric dilation (overload) | |
| | | | pyloric stenosing ulcer | |
| | | | gastric tear following primary overload | |
| | | | gastric tear following enteritis | bacterial |
| | | | | unidentified pathogen |
| | | | | no search for pathogen |
| | | | gastric perforating ulcer | |
| | | | duodenal perforating ulcers | |
| | | | gastric and duodenal perforating ulcer | |
| | | | volvulus | |

| | | | | |
|---|---|---|---|---|
| | | | volvulus nodosus | |
| | | | small intestine entrapment by fibrous strands | |
| | | | intestinal herniation through the epiploic foramen | |
| | | | intussusception of the small intestine | |
| | | | ileo-caecal intussusception | |
| | | | caeco-caecal intussusception | |
| | | | caeco-colic intussusception | |
| | | | torsion of the caecum | |
| | | | torsion of the large colon 180° | |
| | | | torsion of the large colon 360° | |
| | | | renosplenic entrapment | |
| | | | torsion of the caecum and large colon | |
| | | | other | |
| | | | gastric rupture | |
| | | | gastric rupture following stenosing duodenal ulcer | |
| | | | ascending colon - tear | |
| | | | caecal impaction | |
| | | | large colon impaction | |
| | | | dilatation of the digestive reservoirs | |
| | | | caecal infarction | |
| | | | caecum - tear | |
| | | | large colon infarction | |
| | | | entrapment of the small intestine in the mesentery | |
| | | | small intestive infarction | |
| | | | esophageal obstruction | |
| | | toxic | yew | |
| | | | acorn | |
| | | | lead | |
| | | | other | |
| | | | unidentified | |
| | | | right dorsal colitis | |
| | | allergic | | |
| | | metabolic | hyperlipemia | |
| | | | hemosiderosis | |
| | | | other | |
| | unidentified | | | |

| | | | | |
|---|---|---|---|---|
| | other | Grass Sickness | | |
| | | Tyzzer's disease | | |
| | | ileal hypertrophy (idiopathic) | | |
| | | chronic eosinophilic infiltration | | |
| | | liver disease | | |
| | parasitic | intussusception due to *Parascaris equorum* | | |
| | | tear of the small intestine due to *Parascaris equorum* | | |
| | | infarction of the small intestine due to *Strongylus vulgaris* | | |
| | | infarction of the large colon due to *Strongylus vulgaris* | | |
| | | larval cyathostomosis | | |
| | | following deworming | | |
| | | other | | |
| | | tear of the small intestine due to *Anoplocephala spp* | | |
| | | tear of the caecum due to *Anoplocephala spp* | | |
| musculoskeletal system | infectious | osteomyelitis | bacterial | |
| | | | unidentified pathogen | |
| | | | no search for pathogen | |
| | | osteoarticular rhodococcosis | bacterial | |
| | | | unidentified pathogen | |
| | | | no search for pathogen | |
| | | gangrene | anaerobic bacteria | |
| | | | unidentified pathogen | |
| | | | no search for pathogen | |
| | | arthritis | bacterial | |
| | | | unidentified pathogen | |
| | | | no search for pathogen | |
| | | polyarthritis | bacterial | |
| | | | unidentified pathogen | |
| | | | no search for pathogen | |
| | | unidentified | | |
| | | tenosynovitis | bacterial | |
| | | | unidentified pathogen | |
| | | | no search for pathogen | |
| | | suppurative myositis | bacterial | |
| | | | unidentified pathogen | |
| | | | no search for pathogen | |
| | | other | | |

| | | | | |
|---|---|---|---|---|
| | non infectious | tumoral | hemangiosarcoma | |
| | | | osteoma | |
| | | | other | |
| | | allergic | | |
| | | toxic | atypical myopathy | |
| | | | other | |
| | | metabolic | equine polysaccharide storage myopathy | |
| | | | exertional rhabdomyolysis | |
| | | | post-anesthesia myopathy | |
| | | | laminitis | front feet |
| | | | | hind feet |
| | | | | 4 feet |
| | | | degenerative myopathy | |
| | | traumatic | fracture | skull |
| | | | | jaw |
| | | | | limbs-phalanx |
| | | | | limbs-main metacarpal bone |
| | | | | limbs-main metatarsal bone |
| | | | | limbs- elbow |
| | | | | limbs-humerus |
| | | | | lims-tibia |
| | | | | limbs-patella |
| | | | | limbs-femur |
| | | | | pelvis |
| | | | | cervical spine |
| | | | | thoracic spine |
| | | | | other |
| | | | | polytraumatism |
| | | | gastrocnemius muscle rupture | |
| | | | other | |
| | | | tendinous damage | front limb |
| | | | | hind limb |
| | | | cervical trauma | |
| | | | cerebral concussion | |
| | | mechanichal | arthropathy | |
| | | | tendinopathy | |
| | | congenital or hereditary | cervical stenotic myelopathy (Wobbler syndrome) | |
| | | | angular deviation of limbs | |
| | unidentified | | | |
| | other | | | |

| | | | | |
|---|---|---|---|---|
| cardiovascular system | infectious | endocarditis | bacterial | |
| | | | mycotic | |
| | | | unidentified pathogen | |
| | | | no search for pathogen | |
| | | myocarditis | bacterial | |
| | | | mycotic | |
| | | | unidentified pathogen | |
| | | | no search for pathogen | |
| | | pericarditis | bacterial | |
| | | | mycotic | |
| | | | viral | |
| | | | unidentified pathogen | |
| | | | no search for pathogen | |
| | non infectious | tumoral | rhabdomyosarcoma | |
| | | allergic | | |
| | | toxic | | |
| | | traumatic | tamponade | |
| | | cardiogenic shock | acute heart failure | |
| | | | atrial fibrillation | |
| | | | other | |
| | | hemorrhagic shock | rupture of the uterine artery | |
| | | | rupture of the ovarian artery | |
| | | | rupture of the vaginal artery | |
| | | | rupture of the axillary artery | |
| | | | rupture of the aortic arch | |
| | | | rupture of the pulmonary trunk | |
| | | | rupture of the inferior veina cava | |
| | | | rupture of the mesenteric vessels | |
| | | | rupture of the spleen | |
| | | | rupture of the liver | |
| | | | rupture of the wall of the atrium | |
| | | | rupture of the splenic vein | |
| | | | purpura | |
| | | | other | |
| | | | rupture of the testicular artery | |
| | | | the throat has been cut (or throat cutting) | |
| | | | rupture of the renal artery | |
| | | | rupture of the iliac artery | |
| | | | rupture of the brachiocephalic trunk | |
| | | | unidentified | |
| | | congenital abnormality | | |
| | unidentified | | | |
| | other | | | |

| | | | | |
|---|---|---|---|---|
| urinary system and female reproductive organs | infectious | nephritis | bacterial | |
| | | | mycotic | |
| | | | unidentified pathogen | |
| | | | no search for pathogen | |
| | | endometritis | bacterial | |
| | | | unidentified pathogen | |
| | | | no search for pathogen | |
| | non infectious | tumoral | | |
| | | traumatic | uterine tear | |
| | | | uterine hematoma | |
| | | | uterine prolapse | |
| | | | other | |
| | | | perforation of the urinary bladder with prolapse of the large colon | |
| | | | dystocia | |
| | | congenital or hereditary | | |
| | | metabolic | | |
| | | other | renal calculi | |
| | unidentified | | | |
| | other | | | |
| urinary system and male reproductive organs | infectious | orchitis | bacterial | |
| | | | mycotic | |
| | | | viral | |
| | | | unidentified pathogen | |
| | | | no search for pathogen | |
| | | nephritis | bacterial | |
| | | | mycotic | |
| | | | unidentified pathogen | |
| | | | no search for pathogen | |
| | non infectious | tumoral | | |
| | | traumatic | | |
| | | congenital or hereditary | | |
| | | metabolic | | |
| | | other | renal calculi | |
| | unidentified | | | |
| | other | | | |

| | | | | |
|---|---|---|---|---|
| neurologic system | infectious | meningitis | bacterial | |
| | | | mycotic | |
| | | | viral (not herpes) | |
| | | | unidentified pathogen | |
| | | | no search for pathogen | |
| | | myeloencephalopathy | bacterial | |
| | | | mycotic | |
| | | | viral (not herpes) | |
| | | | unidentified pathogen | |
| | | | no search for pathogen | |
| | | guttural pouch mycosis | yeast and fungal | |
| | | | unidentified pathogen | |
| | | | no search for pathogen | |
| | | Rhinopneumonitis (nervous form) | EHV1 | |
| | | other | | |
| | | tetanus | bacterial | |
| | | | unidentified pathogen | |
| | | | no search for pathogen | |
| | | West-Nile Virus | | |
| | | Botulism | bacterial | |
| | | | unidentified pathogen | |
| | | | no search for pathogen | |
| | non infectious | toxic | encephalomalacia | |
| | | | other | |
| | | | hepatic encephalopathy | |
| | | traumatic | damage of the obturator nerve | |
| | | | damage of the brachial plexus | |
| | | | damage of the lumbosacral plexus | |
| | | | other | |
| | | congenital abnormality | | |
| | | tumoral | | |
| | parasitic | Equine protozoal encephalomyelitis | | |
| | unidentified | | | |
| | other | Grass Sickness | | |
| | | Motor neuron disease | | |
| | | myelopathy post anesthesia | | |

| | | | | |
|---|---|---|---|---|
| systemic system | infectious | septicemia | bacterial | |
| | | | unidentified pathogen | |
| | | | no search for pathogen | |
| | | rhodococcosis (pulmonary and digestive form) | bacterial | |
| | | | unidentified pathogen | |
| | | | no search for pathogen | |
| | | rhodococcosis (pulmonary and osteoarticular form) | bacterial | |
| | | | unidentified pathogen | |
| | | | no search for pathogen | |
| | | rhodococcosis (digestive form and osteoarticular form) | bacterial | |
| | | | unidentified pathogen | |
| | | | no search for pathogen | |
| | | rhodococcosis (pulmonary + digestive + osteoarticular form) | bacterial | |
| | | | unidentified pathogen | |
| | | | no search for pathogen | |
| | | Tyzzer's disease | | |
| | | Leptospirosis | | |
| | non infectious | hemorraghic shock | hemorrhagic syndrome or purpura | |
| | | | other | |
| | | anaphylactic shock | vaccine | |
| | | | antibiotic | |
| | | | anesthetic | |
| | | | other | |
| | | electrical shock | lightning | |
| | | | electrocution | |
| | | metabolic | pituitary pars intermedia dysfunction | |
| | | tumoral | lymphosarcoma (or lymphoma) | |
| | | | melanoma | |
| | | heatshock | | |
| | unidentified | | | |
| | other | chronic eosinophilic infiltration | | |
| | | senile | | |
| endocrine system | infectious | | | |
| | non infectious | hyperthyroidism | | |
| | | pituitary pars intermedia dysfunction | | |
| | | tumoral | pheochromocytoma | |
| | unidentified | | | |
| | other | | | |

| | | | | |
|---|---|---|---|---|
| exotic diseases | Rabies | | | |
| | African horse sickness | | | |
| | Eastern equine encephalitis | | | |
| | Western equine encephalitis | | | |
| | Venezuelan equine encephalomyelitis | | | |
| | Japanese encephalitis | | | |
| | Dourine | | | |
| | Vesicular stomatitis | | | |
| | Hendra virus | | | |
| | Aujeszky's disease | | | |
| | Equine epizootic lymphangitis | | | |
| | Glanders | | | |
| | Melioidosis | | | |
| | Pythiosis | | | |
